# Conservative Management of Horizontal Root Fracture: A Case Report

**DOI:** 10.1002/ccr3.9625

**Published:** 2024-12-08

**Authors:** Farid Haghdadi, Amirreza Mokabberi, Seyed Ahmad Banihashem Rad

**Affiliations:** ^1^ North Khorasan University of Medical Sciences Bojnurd North Khorasan Iran; ^2^ Student Research Committee, School of Dentistry Mashhad University of Medical Sciences Mashhad Iran; ^3^ Department of Endodontics, School of Dentistry, Isfahan (Khorasgan) Branch Islamic Azad University Isfahan Iran; ^4^ Department of Restorative, Preventive and Pediatric Dentistry University of Bern Bern Switzerland; ^5^ Graduate School for Health Sciences University of Bern Bern Switzerland

**Keywords:** dental trauma, endodontics, horizontal root fracture, MTA

## Abstract

In cases where permanent teeth have closed apices and completely erupted roots, the probability of root fractures is increased due to the stable support given by the adjacent bone and periodontal tissues. Fractures have the potential to affect several dental structures, including the pulp, dentin, cementum, bone, and periodontal tissues. In cases of horizontal root fractures (HRF), the apical section typically maintains vitality; however, the present case presented an unusual apex necrosis. Given the patient's age and tooth stability, traditional apical surgery was deemed inappropriate. Instead, a conservative treatment approach was chosen to preserve the tooth. This case report details the management and a 2‐year follow‐up of a maxillary central incisor exhibiting a HRF and apical necrosis, successfully addressed through the application of Mineral Trioxide Aggregate (MTA) in the both apical and coronal segments. Using MTA prevented apical surgery in cases of apex necrosis accompanied by HRF.

**Summary**

- Our study showed the effectiveness of Mineral Trioxide Aggregate (MTA) as a conservative treatment option for horizontal root fractures with apical necrosis in fully erupted permanent teeth.
- The efficacy of MTA was demonstrated in both apical and coronal segments over the course of a 2‐year follow‐up study, demonstrating its potential as an alternative to traditional apical surgery in challenging clinical situations.

## 1 Introduction

Root fractures, which encompass fractures affecting dentine, cementum, and pulp, account for only 0.5%–7% of dental injuries. Among these injuries, individuals aged 10–20 years exhibit the highest susceptibility [1]. The occurrence of horizontal root fractures (HRF) is primarily reported in the anterior region of the maxilla with approximately 75% of cases involved the maxillary central incisors, primarily affecting male patients due to trauma resulting from car accidents, sports injuries, fights, and similar incidents [1]. Root fractures are more commonly observed in fully erupted permanent teeth that have closed apices, meaning that the root completely formed and firmly supported by the surrounding bone and periodontium [2]. The potential outcomes can exhibit complexity due to the cumulative damage of the pulp, dentin, cementum, bone, and periodontium.

The term “horizontal root fracture” (HFR) refers to a type of injury that affects multiple components of the tooth, including the pulp, dentin, cementum, and periodontal ligament [3]. The positioning of the fracture line may exhibit variability, ranging from the coronal and middle third to the apical third. The displacement of the coronal portion of the tooth is an often observed phenomenon, although involvement of the apical section is rare [4, 5]. Some studies have confirmed that healing along the fracture line in cases of HRF typically exhibits a favorable prognosis following relocation and the application of a flexible splint [6, 7].

The healing process is influenced by various factors, including the patient's age, tooth mobility, degree of root formation, location of root fracture, diastasis of fragments, and the duration between the occurrence of trauma and the initiation of treatment [7, 8].

Mineral Trioxide Aggregate (MTA) is a biocompatible material that contains a number of clinical applications within the field of endodontics. The material exhibits potential advancements compared to alternative materials in some endodontic operations pertaining to root repair and healing of the bone [9, 10, 11].

This case report describes a rare occurrence of apical necrosis followed after a HRF in a maxillary central incisor. This case is unique in that the treatment approach involved the placement of MTA in both apical and coronal segments, which differs from the traditional approach of performing apical surgery.

## 2 Case History/Examination

**FIGURE 1:**
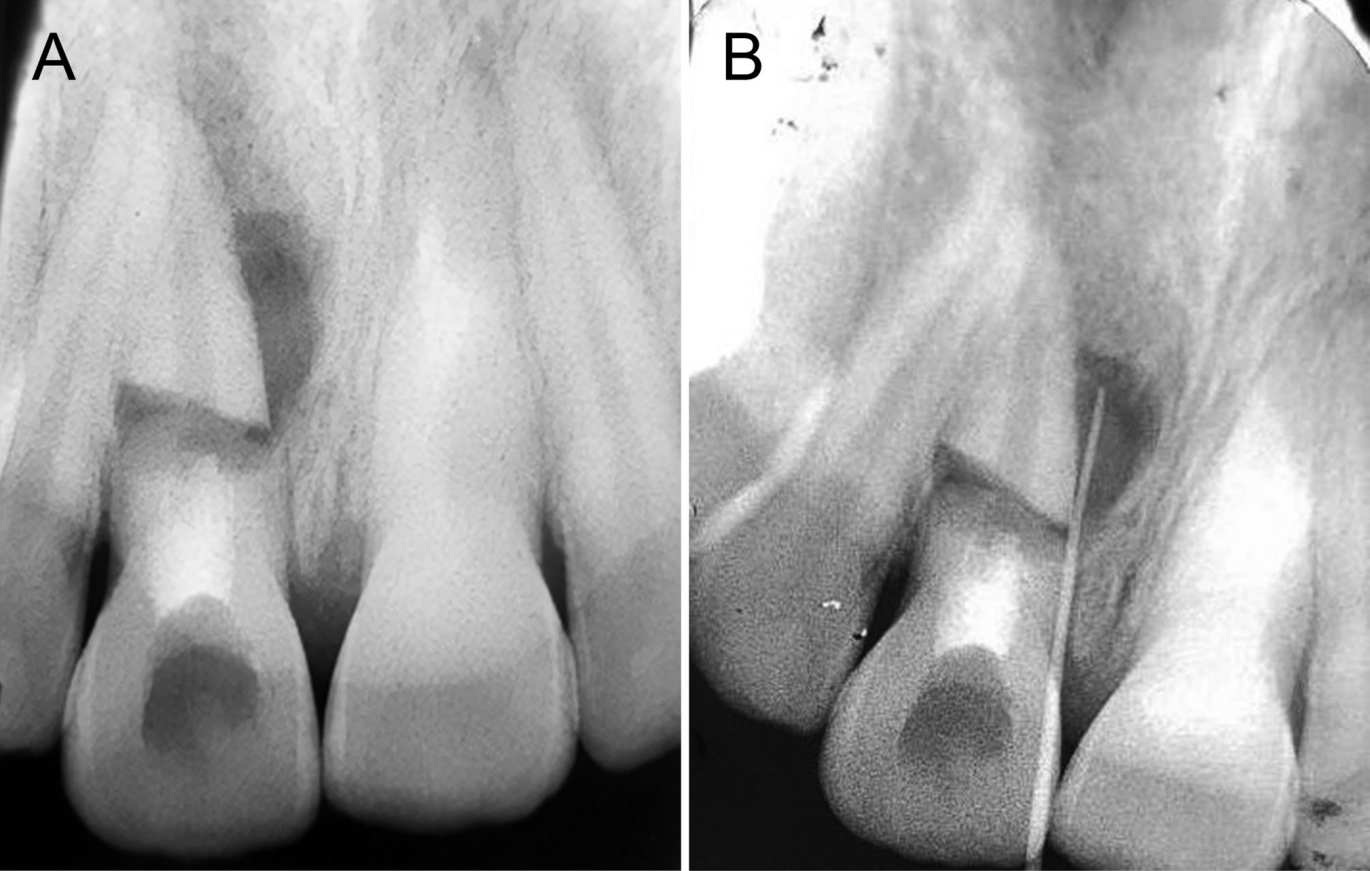
(A) Tooth #8 with the existing lesion. The coronal segment was filled by MTA in the previous intervention. (B) The sinus tract is traced by gutta‐percha.

The pediatric department of Mashhad Dental School referred a 14‐year‐old male patient with mild pain in the anterior maxilla to the endodontic department. The patient's medical history revealed a traumatic incident 5 years prior involving a car accident, which resulted in a HRF in the right maxillary central incisor (#8). Initial clinical examinations included assessment of the tooth's mobility, tenderness to percussion, and presence of sinus tracts or swelling. Observations revealed Grade II mobility in tooth #8 as well as a persistent sinus tract, which is indicative of possible apical periodontitis. An examination of the probing depth indicated a depth of 6 mm, and clinical signs suggested ongoing inflammation. Initial clinical and radiographic examinations revealed the presence of this fracture without segment displacement (Figure 1A). The patient had previously undergone an intervention in the pediatric department, where the coronal segment of the tooth was sealed with MTA. However, a persistent lesion in the preapical radiograph and a sinus tract near the mucogingival line persisted after the treatment, indicating apical periodontitis (probing depth 6 mm). The tooth showed Grade II mobility(> 1 mm) according to Miller's classification of tooth mobility [12]. The sinus tract was then traced using gutta‐percha #25 (Figure 1B).

Determination of the vitality of the apical root segment was a crucial factor in the treatment plan. The coronal portion of the crown could be re‐disinfected and sealed with traditional silicate cements if the apex was vital. If the apex was found to be necrotic, the surgical extraction of the apical segment was the first conventional approach. Therefore, the previously applied MTA should be removed from the coronal segment in order to determine the vitality of the apical segment.

All anterior teeth, including tooth #9, were thoroughly examined, and tooth #9 was closely monitored for any changes. Apical radiographs showed canal calcification and potential apical widening. In spite of the fact that tooth #9 required no immediate intervention, follow‐up appointments ensured comprehensive care and early detection of any problems.

## 3 Methods (Differential Diagnosis, Investigations, and Treatment)

In the first visit, local anesthesia was administered 2% lidocaine with 1:100,000 epinephrine (Xylestesin‐A 2%, 3 M ESPE, Seefeld, Germany), and a rubber dam was used to isolate the tooth. The root canal was accessed using a Zeiss surgical microscope (Oberkochen, Germany) and gently irrigated with a 2.5% sodium hypochlorite (NaOCl) solution. To remove the previously applied MTA from the coronal segment, mechanical devices, such as ultrasonic instruments, mueller burs, and 37% hydrochloric acid (HCl), were utilized. MTA removal was facilitated by using 37% HCl, which reduced microhardness and push‐out bond strength [10]. Calcium hydroxide medication was applied to the coronal part of the root canal, and a two‐week follow‐up appointment was scheduled.

Due to the lesion's extension in the apical, lateral, and coronal directions, it was uncertain if the apical section was necrotic. Penetrating the apical segment may have jeopardized the tooth's stability. The coronal portion, sealed with MTA, acted as a temporary barrier. This allowed us to observe the tooth's reaction and determine if the lesion would resolve naturally before considering more invasive therapies. If there is no evidence of healing, we would then proceed with treating the apical section.

**FIGURE 2:**
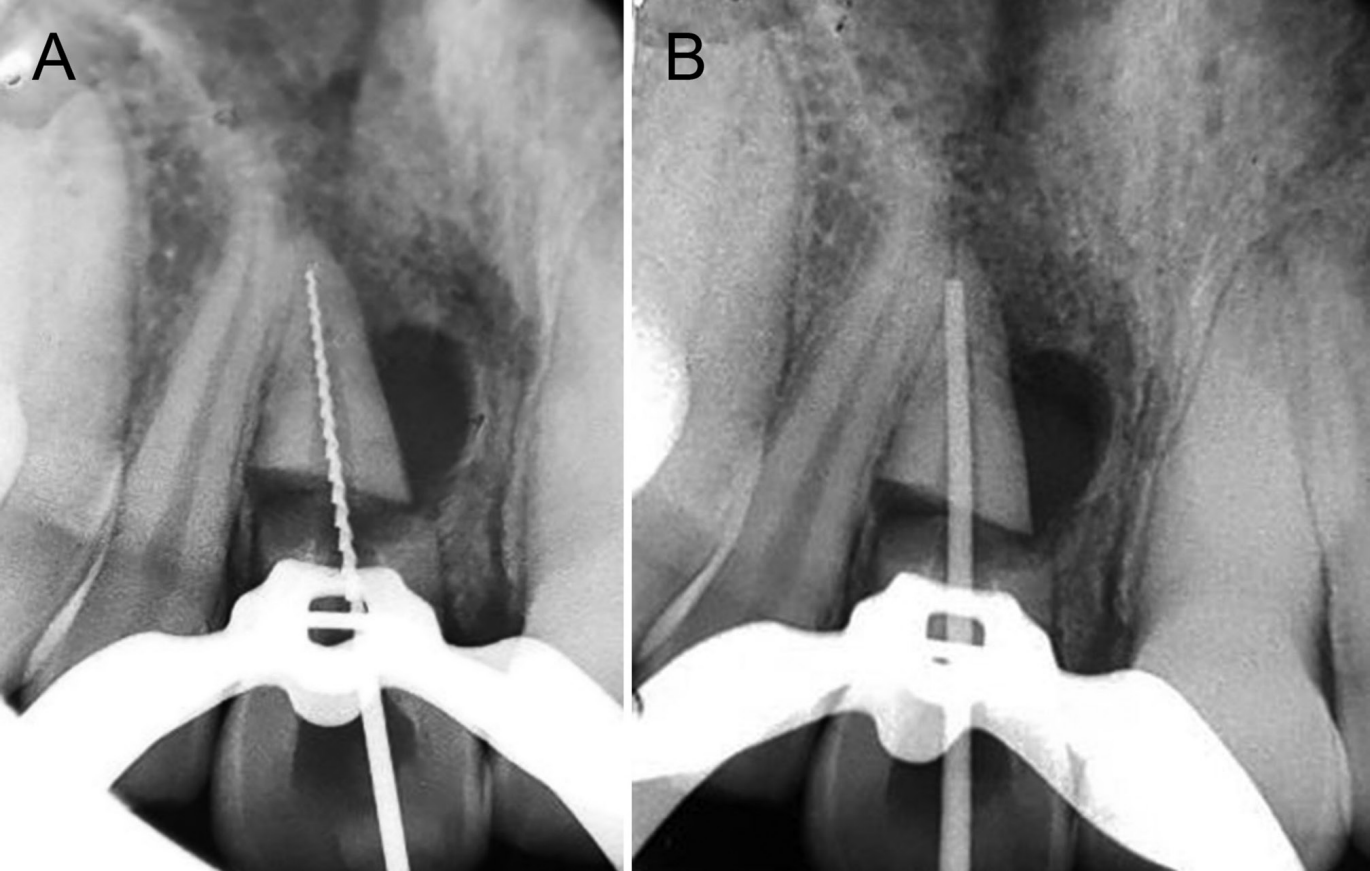
Using #20 Hand K file for measuring the length of the root (A) gutta‐percha (B).

At the second dental visit, persistent symptoms and the presence of a sinus tract in tooth #8 confirmed the necrosis of the apical segment, which required treatment similar to that of the coronal segment. Due to concerns about compromising tooth stability and potential tooth loss and considering the age of the patient, surgical intervention was ruled out. Therefore, the treatment plan was adjusted to concentrate on cleaning and sealing the apical segment with silicate cements. As part of this procedure, local infiltration anesthesia with 2% lidocaine was administered, the tooth was isolated with a rubber dam, and the access cavity was reopened. Metapaste dressing was gently removed from the coronal canal space using NaOCl, followed by a sterile saline solution rinse. Afterward, the coronal canal was meticulously rinsed with a 17% EDTA solution (Pulpdent, Watertown, MA) and dried. Based on the periapical radiograph, the tooth's length was determined to be 18 mm from the incisor edge to the radiographic apex. To shape the canal, we used a hand K‐file #20 measuring 18 mm to penetrate the apical fragment delicately. Following irrigation with 1% NaOCl, the canal was dried with paper points (Figure 2).

**FIGURE 3:**
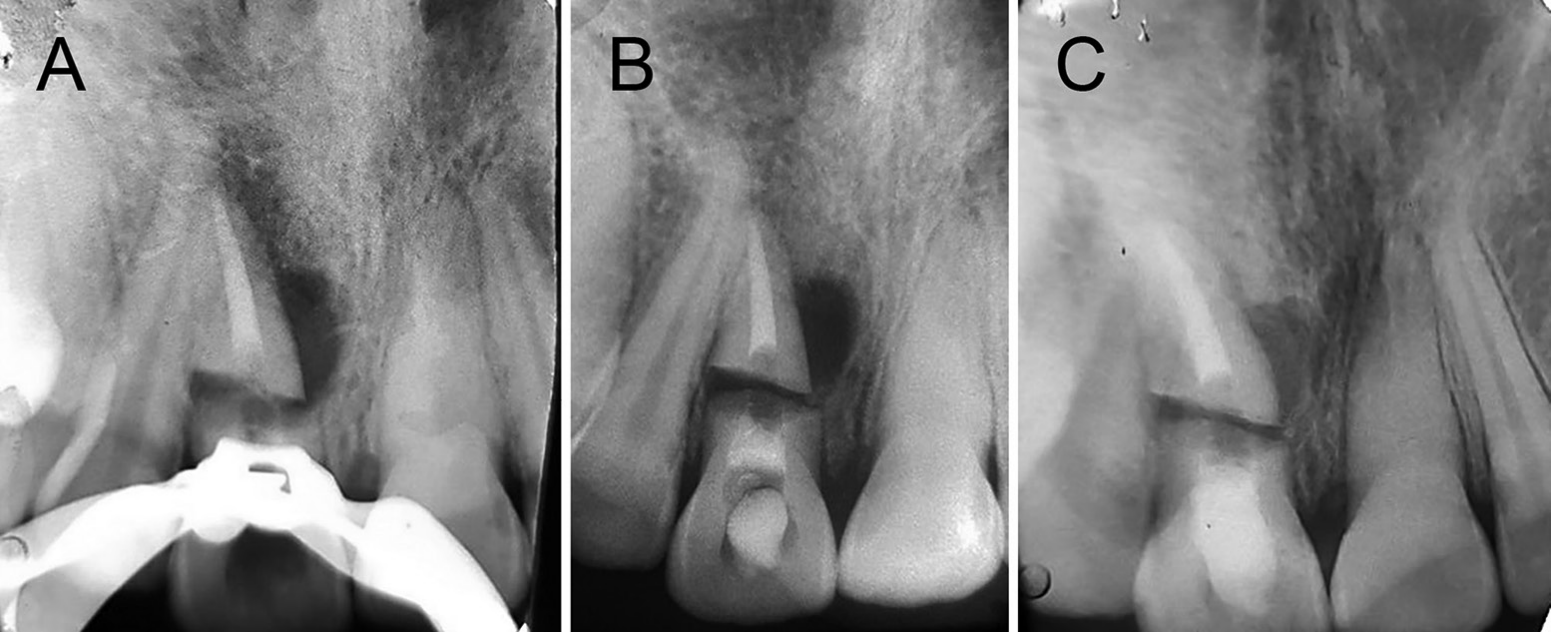
(A) Obturation of apical segment by MTA. (B) Obturation of both apical and coronal fragment by MTA. Using collaplug between two segments in the third treatment session. (C) Healing lesion after two years follow‐up.

We used an apex locator (Morita Root ZX II) to precisely measure the length of apical fragment, minimizing the risk of inadvertently pushing MTA into the space between the coronal and apical segments. A MTA carrier was used to obturate the apical segment with MTA (Figure 3A). After this, a moistened cotton pellet was inserted into the coronal segment, and the access cavity was sealed with Cavit and glass ionomer cement. Two weeks later, the patient was scheduled for a follow‐up appointment (Figure 3A).

In the next appointment, the patient showed no symptoms, and the previously observed sinus tract had fully healed. After administering local anesthesia with 2% lidocaine, the tooth was isolated with a rubber dam. The access cavity was reopened, and the coronal part of the canal was rinsed with 5 mL of 5.25% NaOCl. The integrity of the MTA seal was confirmed. CollaPlug fibers (Zimmer Dental, Carlsbad, CA) were placed between the two segments, and the coronal segment was sealed with MTA (Figure 3B). A moist cotton pellet was applied over the MTA, and the tooth was temporized. A week later, the MTA setting was confirmed and a composite restoration was applied to the patient's teeth.

## 4 Conclusion and Results (Outcome and Follow‐Up)

At the 2‐year follow‐up, the patient was asymptomatic, and clinical examination revealed no signs of edema, erythema, or sinus tracts. The tooth #8 waas not sensitive to percussion and palpation, and all probing depths were within normal ranges. According to radiographic examination, the radiolucent lesions on tooth #8 have healed with fibrosis tissue (Figure 3C). The radiograph obtained 2 years afterward (Figure 3C) reveals the presence of canal calcification in tooth number #7. The calcification found is a favorable outcome of the trauma, suggesting the tooth's natural healing reaction and ongoing vitality.

It is possible to avoid apical surgery in instances of apex necrosis accompanied by HRF through the use of MTA. MTA was used to fill the fractured apical segment of the root. Following a 24‐month follow‐up examination, the patient was asymptomatic and had excellent healing patterns on both clinical and radiographic level. This suggests that MTA can be a favorable choice for a definitive root filling material in horizontal fractures due to its exceptional biological and physical properties.

## 5 Discussion

Due to the relatively high incidence of necrosis in horizontal root fractures, it has been recommended that immediate endodontic intervention be avoided in these cases, and that clinical and radiographic follow‐up should be the treatment of choice, provided no clinical and/or pathological signs are present [13]. In reported case as there was clinical signs, the endodontic treatment was performed.

In fractured teeth with pulp necrosis, the apical part remains vital. Therefore, root canal therapy is typically limited to the coronal segment [1]. In the present case, however, the apex was found to be necrotic, which is unusual. Due to patient's age and the tooth's stability, traditional apical surgery was not recommended. As a result, we decided on a more conservative treatment plan to preserve the tooth.

Conventional endodontic treatment typically targets the coronal fragment exclusively in cases of apical and middle‐third fractures. The coronal canal is filled with gutta‐percha, and a stop is prepared using non‐setting calcium hydroxide or MTA. The use of calcium hydroxide in the past has been associated with a number of drawbacks, such as the need for multiple applications, the possibility of reinfection, and the potential for root fractures in immature teeth [14, 15]. Therefore, MTA is recommended for teeth with necrotic pulps and open apices [16]. MTA provides several advantages over calcium hydroxide, including increased fracture resistance, improved success in achieving apical closure, reduced failure rates, enhanced hard tissue formation, and reduced inflammation [17, 18]. In the present case, MTA was selected for root canal treatment in order to improve treatment outcomes [19].

In spite of its remarkable biological performance, MTA has several clinical drawbacks, including a discoloration potential and difficulty in removing it after setting, making it difficult to retreat a failed case in a fractured tooth [20]. The application of White MTA was employed in this case to mitigate aesthetic concerns and ensure optimal results in terms of both appearance and functionality. The coronal segment did not show any visible change in color after treatment, demonstrating that white MTA is a suitable material for preserving the natural appearance of the tooth while also offering successful treatment [21].

Radiographic detection of horizontal root fractures requires proper angulation of X‐rays. It is recommended to take multiple radiographs from various angles when suspecting a horizontal root fracture; therefore different preapical radiographs were taken at various angles [10]. In the present case, the periapical radiograph revealed a horizontal root fracture without significant displacement of the segments. At two‐week and two‐year intervals, follow‐up radiographs were taken to monitor healing and assess the apical and coronal segments. In the two‐year follow‐up radiograph, the radiolucent lesions had significantly resolved, and the fracture site had healed with fibrous tissue.

Root fractures in teeth can undergo several healing mechanisms, such as direct bone healing, connective tissue healing, or the development of fibrous tissue. Several factors contribute to fibrous tissue healing, including delayed treatment, significant displacement of fracture segments, and poor periodontal tissue health. Because fibrous tissue does not restore the tooth's original strength or functionality, it is generally less favorable than bone or hard tissue healing. As a result of the lack of mineralization associated with bone healing, fibrous tissue repairs tend to be weaker and less stable. There is a potential for reinfection or further damage to teeth as a result of persistent clinical issues such as tooth mobility. The formation of mineralized tissue during bone or hard tissue healing results in a more efficient restoration of tooth structure and function, which reduces the risk of long‐term complications [22, 23, 24].

The present case report illustrates the success of MTA in the treatment of root‐fractured teeth with apical pulp necrosis.

## Author Contributions

**Farid Haghdadi:** conceptualization, project administration, supervision, writing – review and editing. **Amirreza Mokabberi:** investigation, writing – review and editing. **Seyed Ahmad Banihashem Rad:** conceptualization, project administration, supervision, writing – original draft, writing – review and editing.

## Consent

Written informed consent was obtained from the patient for the publication of the case report and the accompanying images.

## Conflicts of Interest

The authors declare no conflicts of interest.

## Data Availability

Data sharing is not applicable to this article as no new data were created or analyzed in this study.

## References

[1] J. O. Andreasen , F. M. Andreasen , and L. Andersson , Textbook and Color Atlas of Traumatic Injuries to the Teeth, vol. 6 (Hoboken, NJ: John Wiley & Sons, 2018), 279.

[2] A. Majorana , S. Pasini , E. Bardellini , and E. Keller , “Clinical and Epidemiological Study of Traumatic Root Fractures,” Dental Traumatology 18, no. 2 (2002): 77–80. 12184216 10.1034/j.1600-9657.2002.180206.x

[3] E. Kucukyilmaz , M. S. Botsali , and G. Keser , “Treatments of Horizontal Root Fractures: Four Case Reports,” Journal of Pediatric Dentistry 1, no. 1 (2013): 19–23.

[4] F. Andreasen , “Examination and Diagnosis of Dental Injuries,” in Textbook and Color Atlas of Traumatic Injuries to the Teeth, eds. J. O. Andreasen , F. M. Andreasen , and L. Andersson (Oxford: Blackwell, 2007).

[5] M. Calişkan and Y. Pehlivan , “Prognosis of Root‐Fractured Permanent Incisors,” Dental Traumatology 12, no. 3 (1996): 129–136. 10.1111/j.1600-9657.1996.tb00111.x 9028190

[6] J. Andreasen , F. Andreasen , I. Mejare , and M. Cvek , “Healing of 400 Intra‐Alveolar Root Fractures. 2. Effect of Treatment Factors Such as Treatment Delay, Repositioning, Splinting Type and Period and Antibiotics,” Dental Traumatology 20, no. 4 (2004): 203–211. 15245519 10.1111/j.1600-9657.2004.00278.x

[7] N. Sisodia and M. Manjunath , “Conservative Management of Horizontal Root Fracture–A Case Series,” Journal of Clinical and Diagnostic Research: JCDR 9, no. 8 (2015): ZD04. 10.7860/JCDR/2015/12959.6284 PMC4576655 26436061

[8] J. Andreasen , F. Andreasen , I. Mejare , and M. Cvek , “Healing of 400 Intra‐Alveolar Root Fractures. 1. Effect of Pre‐Injury and Injury Factors Such as Sex, Age, Stage of Root Development, Fracture Type, Location of Fracture and Severity of Dislocation,” Dental Traumatology 20, no. 4 (2004): 192–202. 15245518 10.1111/j.1600-9657.2004.00279.x

[9] R. S. Schwartz , M. Mauger , D. J. Clement , and W. A. Walker, III , “Mineral Trioxide Aggregate: A New Material for Endodontics,” Journal of the American Dental Association 130, no. 7 (1999): 967–975. 10422400 10.14219/jada.archive.1999.0337

[10] C. M. Bramante , R. Menezes , I. G. Moraes , N. Bernardinelli , R. B. Garcia , and A. Letra , “Use of MTA and Intracanal Post Reinforcement in a Horizontally Fractured Tooth: A Case Report,” Dental Traumatology 22, no. 5 (2006): 275–278. 16942558 10.1111/j.1600-9657.2006.00353.x

[11] S. Staffoli , G. Plotino , B. G. Nunez Torrijos , et al., “Regenerative Endodontic Procedures Using Contemporary Endodontic Materials,” Materials 12, no. 6 (2019): 908. 30893790 10.3390/ma12060908 PMC6471897

[12] C.‐P. Wu , Y.‐K. Tu , S.‐L. Lu , J.‐H. Chang , and H.‐K. Lu , “Quantitative Analysis of Miller Mobility Index for the Diagnosis of Moderate to Severe Periodontitis—A Cross‐Sectional Study,” Journal of Dental Sciences 13, no. 1 (2018): 43–47. 30895093 10.1016/j.jds.2017.11.001 PMC6388838

[13] F. M. Andreasen and B. V. Pedersen , “Prognosis of Luxated Permanent Teeth—The Development of Pulp Necrosis,” Dental Traumatology 1, no. 6 (1985): 207–220. 10.1111/j.1600-9657.1985.tb00583.x 3867505

[14] J. O. Andreasen , B. Farik , and E. C. Munksgaard , “Long‐Term Calcium Hydroxide as a Root Canal Dressing May Increase Risk of Root Fracture,” Dental Traumatology 18, no. 3 (2002): 134–137. 12110105 10.1034/j.1600-9657.2002.00097.x

[15] J. O. Andreasen , E. C. Munksgaard , and L. K. Bakland , “Comparison of Fracture Resistance in Root Canals of Immature Sheep Teeth After Filling With Calcium Hydroxide or MTA,” Dental Traumatology 22, no. 3 (2006): 154–156. 16643291 10.1111/j.1600-9657.2006.00419.x

[16] A. Kusgoz , T. Yildirim , M. Tanriver , and C. Yesilyurt , “Treatment of Horizontal Root Fractures Using MTA as Apical Plug: Report of 3 Cases,” Oral Surgery, Oral Medicine, Oral Pathology, Oral Radiology, and Endodontology 107, no. 5 (2009): e68–e72. 10.1016/j.tripleo.2009.01.031 19426911

[17] Š. Hatibović‐Kofman , L. Raimundo , L. Zheng , L. Chong , M. Friedman , and J. O. Andreasen , “Fracture Resistance and Histological Findings of Immature Teeth Treated With Mineral Trioxide Aggregate,” Dental Traumatology 24, no. 3 (2008): 272–276. 18410392 10.1111/j.1600-9657.2007.00541.x

[18] M. Torabinejad , M. Parirokh , and P. M. Dummer , “Mineral Trioxide Aggregate and Other Bioactive Endodontic Cements: An Updated Overview–Part II: Other Clinical Applications and Complications,” International Endodontic Journal 51, no. 3 (2018): 284–317. 28846134 10.1111/iej.12843

[19] S. Shabahang , M. Torabinejad , P. P. Boyne , H. Abedi , and P. McMillan , “A Comparative Study of Root‐End Induction Using Osteogenic Protein‐1, Calcium Hydroxide, and Mineral Trioxide Aggregate in Dogs,” Journal of Endodontics 25, no. 1 (1999): 1–5. 10196834 10.1016/S0099-2399(99)80388-4

[20] M. Forghani , M. Bidar , F. Shahrami , M. Bagheri , M. Mohammadi , and N. A. Mashhadi , “Effect of MTA and Portland Cement on Fracture Resistance of Dentin,” Journal of Dental Research, Dental Clinics, Dental Prospects 7, no. 2 (2013): 81. 23875085 10.5681/joddd.2013.014 PMC3713865

[21] A. Salem‐Milani , S. Ghasemi , S. Rahimi , A. Ardalan‐Abdollahi , and M. Asghari‐Jafarabadi , “The Discoloration Effect of White Mineral Trioxide Aggregate (WMTA), Calcium Enriched Mixture (CEM), and Portland Cement (PC) on Human Teeth,” Journal of Clinical and Experimental Dentistry 9, no. 12 (2017): e1397–e1401. 29410754 10.4317/jced.54075 PMC5794116

[22] A. O. G. S. Heithersay and B. Kahler , “Healing Responses Following Transverse Root Fracture: A Historical Review and Case Reports Showing Healing With (a) Calcified Tissue and (b) Dense Fibrous Connective Tissue,” Dental Traumatology 29, no. 4 (2013): 253–265. 23331373 10.1111/edt.12029

[23] C. Yu and P. V. Abbott , “Responses of the Pulp, Periradicular and Soft Tissues Following Trauma to the Permanent Teeth,” Australian Dental Journal 61 (2016): 39–58. 26923447 10.1111/adj.12397

[24] J. O. Andreasen , S. S. Ahrensburg , and G. Tsilingaridis , “Root Fractures: The Influence of Type of Healing and Location of Fracture on Tooth Survival Rates—An Analysis of 492 Cases,” Dental Traumatology 28, no. 5 (2012): 404–409. 22443169 10.1111/j.1600-9657.2012.01132.x

